# Estimating right atrial pressure using upright computed tomography in patients with heart failure

**DOI:** 10.1007/s00330-022-09360-8

**Published:** 2022-12-28

**Authors:** Ryoma Fukuoka, Yoshitake Yamada, Masaharu Kataoka, Yoichi Yokoyama, Minoru Yamada, Keiichi Narita, Takehiro Nakahara, Keiichi Fukuda, Masahiro Jinzaki

**Affiliations:** 1 Department of Cardiology, Keio University School of Medicine, 35 Shinanomachi, Shinjuku-ku, Tokyo, Japan; 2 Department of Cardiology, School of Medicine, International University of Health and Welfare, 4-3, Kozunomori, Narita, Chiba, Japan; 3 Department of Radiology, Keio University School of Medicine, 35 Shinanomachi, Shinjuku-ku, Tokyo, Japan; 4 The Second Department of Internal Medicine, University of Occupational and Environmental Health, 1-1 Iseigaoka, Yahatanishi-ku, Kitakyushu, Fukuoka, Japan

**Keywords:** Heart failure, Atrial pressure, Superior vena cava, Standing position, Multidetector computed tomography

## Abstract

**Objectives:**

Upright computed tomography (CT) can detect slight changes particularly in the superior vena cava (SVC) volume in healthy volunteers under the influence of gravity. This study aimed to evaluate whether upright CT-based measurements of the SVC area are useful for assessing mean right atrial pressure (mRAP) in patients with heart failure.

**Methods:**

We performed CT in both standing and supine positions to evaluate the SVC (directly below the junction of the bilateral brachiocephalic veins) and inferior vena cava (IVC; at the height of the diaphragm) areas and analyzed their relationship with mRAP, measured by right heart catheterization in 23 patients with heart failure.

**Results:**

The median age of enrolled patients was 60 (51−72) years, and 69.6% were male. The median mRAP was 3 (1−7) mmHg. The correlations between the standing position SVC and IVC areas and mRAP were stronger than those in the supine position (SVC, *ρ* = 0.68, *p* < 0.001 and *ρ* = 0.43, *p* = 0.040; IVC, *ρ* = 0.57, *p* = 0.005 and *ρ* = 0.46, *p* = 0.026; respectively). Furthermore, the SVC area in the standing position was most accurate in identifying patients with higher mRAP (> 5 mmHg) (SVC standing, area under the receiver operating characteristic curve [AUC] = 0.91, 95% confidence interval [CI], 0.77–1.00; SVC supine, AUC = 0.78, 95% CI, 0.59–0.98; IVC standing, AUC = 0.77, 95% CI, 0.55–0.98; IVC supine, AUC = 0.72, 95% CI, 0.49–0.94). The inter- and intraobserver agreements (evaluated by intraclass correlation coefficients) for all CT measurements were 0.962–0.991.

**Conclusions:**

Upright CT-based measurement of the SVC area can be useful for non-invasive estimation of mRAP under the influence of gravity in patients with heart failure.

**Key Points:**

*• This study showed that the superior vena cava (SVC) area in the standing position was most accurate in identifying patients with heart failure with higher mean right atrial pressure.*

*• Upright computed tomography-based measurements of the SVC area can be a promising non-invasive method for estimating mean right atrial pressure under the influence of gravity in patients with heart failure.*

*• Clinical management of patients with heart failure based on this non-invasive modality may lead to early assessment of conditional changes and reduced hospitalization for exacerbation of heart failure.*

**Supplementary Information:**

The online version contains supplementary material available at 10.1007/s00330-022-09360-8.

## Introduction

Recently, a 320-detector-row upright computed tomography (CT) was developed; this novel imaging device permits non-invasive three-dimensional assessment of the entire human body in standing or sitting position [1]. It has been previously reported that upright CT can potentially detect slight changes in vena cava volume in healthy volunteers [1]. In particular, upright CT can quantitatively detect changes in the superior vena cava (SVC) under the influence of gravity [1]. This implies that upright CT can be used as a hemodynamic assessment modality, whereas chest X-ray can be performed in the upright position; however, it does not allow an accurate evaluation of the cardiopulmonary circulation system three-dimensionally [1–3]. Furthermore, echocardiography can quantitatively measure the inferior vena cava (IVC); however, it is usually performed in the supine position without the influence of gravity, and cannot measure the SVC. These findings suggest the potential of upright CT for assessing the hemodynamic profile in diseases in which the influence of gravity should be considered when evaluating a patient’s condition.

Symptoms of dyspnea, such as orthopnea and paroxysmal nocturnal dyspnea, are often considered as specific symptoms in patients with heart failure (HF) and are influenced by patient positioning [4, 5], suggesting the importance of hemodynamic assessment with careful consideration of the influence of gravity when evaluating patients with HF. Assessment of hemodynamic parameters is essential for the diagnosis and optimization of medical therapy in HF patients [6, 7], and mean right atrial pressure (mRAP) is a simple and objective index of intravascular volume status [8, 9]. The IVC diameter and its collapsibility index determined by two-dimensional echocardiography to estimate mRAP are commonly used in clinical settings [8, 9]. However, the accuracy of these parameters is limited when estimating mRAP in an individual patient [8–11]. Right heart catheterization (RHC) is the gold standard for hemodynamic diagnosis; however, it is an invasive method and carries procedural risks [6]. Thus, novel methods for assessing mRAP would be needed.

We hypothesized that upright CT may be useful for assessing hemodynamics, particularly upright CT-based measurements of the SVC area, and could estimate mRAP measured by RHC, under the influence of gravity in patients with HF. Therefore, the present study aimed to investigate the changes in the SVC and IVC areas detected by upright CT as compared with those detected by conventional CT in the supine position, and elucidate the correlation between those areas measured by both CT modalities and mRAP measured by RHC.

## Materials and methods

### Ethics approval

This cross-sectional study was conducted in accordance with the amended Declaration of Helsinki and with the approval of our institutional review board. Written informed consent was obtained from all patients.

### Study population

Patients with a current or past history of HF (Stage C/D determined according to the American College of Cardiology/American Heart Association guidelines [12]) who underwent RHC in our single university-based hospital center from October 2017 to March 2020 were prospectively included if they were able to undergo both upright and conventional CT scans with consideration of their schedules and clinical conditions. The diagnosis of HF was made by experienced attending cardiologists based on the Framingham study criteria [13]. Both CTs were performed within 1 day of RHC if the patients could maintain a standing position safely during the examination, without oxygen therapy. The exclusion criteria were as follows: (i) a recent history of syncope or severe arrhythmia, (ii) renal replacement therapy, (iii) congenital heart disease, (iv) a history of thoracic surgery, and (v) implanted pacemaker or defibrillator. Finally, 23 consecutive patients with HF who underwent RHC, as well as CT scans within 1 day, were enrolled.

### CT imaging protocol

**Fig. 1:**
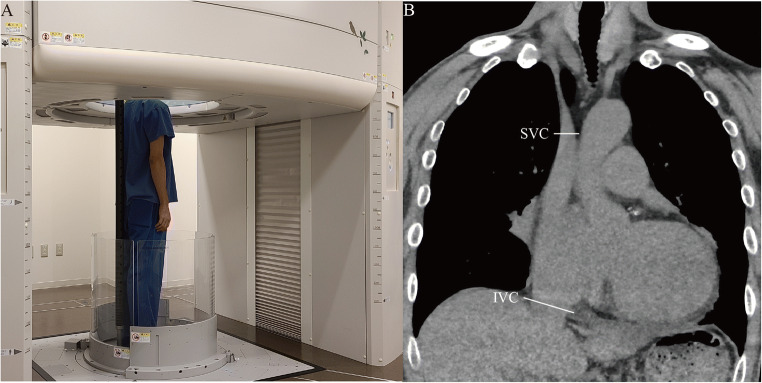
Upright computed tomography (CT) machine and measurements of the superior vena cava (SVC) and inferior vena cava (IVC) areas. **A** Upright CT was performed in the standing position with the subject’s arms down during deep inspiration breath-hold. The upright CT system allows up-and-down movement of a transverse 320-detector-row gantry. **B** Measured points on the vena cava: the SVC was measured directly below the junction of the bilateral brachiocephalic veins, and the IVC was measured at the height of the diaphragm

All patients underwent both upright chest low-radiation-dose CT in the standing position, with arms down, using a 320-detector-row upright CT (prototype TSX-401R, Canon Medical Systems; Fig. 1A) [1–3, 14–17], and conventional chest low-radiation-dose CT in the supine position, with arms raised, using a 320-detector-row CT (Aquilion ONE, Canon Medical Systems). These two examinations were performed consecutively within 2 hours; both scans were unenhanced and were performed during deep inspiration breath-hold, with automatic exposure control using a noise index of 24 for a 5-mm slice thickness (tube current range, 10–350 mA). Other scanning parameters were also the same for both scans: peak tube voltage, 120 kVp; rotation speed, 0.5 seconds; slice collimation, 0.5 mm × 80; and pitch factor, 0.813. The series of contiguous 0.5-mm-thick images was reconstructed with Adaptive Iterative Dose Reduction 3D (Canon Medical Systems) [18].

### CT-based measurements

Measurements of the SVC and IVC areas were manually performed by tracing the edges of the vessels freehand on orthogonal cross-sections of the target vessel using a commercially available workstation (Vitrea, Canon Medical Systems). The SVC area was measured directly below the junction of the bilateral brachiocephalic veins [1], and the IVC area at the height of the diaphragm [1, 19], as previously described (Fig. 1B).

For all patients with HF, the first measurement was performed by a cardiologist with 11 years of experience. A second measurement was performed by the same reader at 1 month after the first reading to assess intraobserver agreement. To assess interobserver agreement, measurements were performed by a general radiologist with 7 years of experience. All measurements were performed in a blinded and randomized manner.

### Right heart catheterization

All patients had a clinical indication for RHC based on HF guidelines [20]. One of three cardiologists with 11, 10, and 9 years of experience, specializing in cardiovascular intervention, performed the RHC. RHC was performed in the supine position, using the Swan−Ganz catheter. Data were acquired through automated measurements of typical pressure waveforms averaged throughout the respiratory cycle.

### Statistical analysis

Continuous variables were presented as medians and 25th–75th percentiles, and categorical variables were presented as absolute values and percentages. Continuous variables were compared using Wilcoxon’s signed-rank test and categorical variables by Pearson’s chi-square test. Inter- and intraobserver agreements were evaluated by calculating intraclass correlation coefficients. Correlations between the CT-based and RHC measurements were determined using Spearman’s correlation coefficient. The strength of the correlation was defined as follows: weak, 0 ≤ |*ρ*| < 0.3; moderate, 0.3 ≤ |*ρ*| < 0.7; or strong, 0.7 ≤ |*ρ*| ≤ 1.0. Discrimination of the CT-based measurements was assessed by calculating the area under the receiver operating characteristic curve (AUC) for normal versus high mRAP (> 5 mmHg). An AUC ≥ 0.6 was considered moderate, an AUC > 0.7 was considered reasonable, and an AUC > 0.8 was considered strong. Bland−Altman plots were generated to evaluate the agreement between upright and supine measurements of the SVC and IVC areas. *p* values < 0.05 were considered to indicate statistical significance. All statistical analyses were performed using SPSS version 26 (SPSS Inc., IBM Corp).

## Results

### Patient characteristics

The clinical characteristics and hemodynamic parameters of 23 patients with HF are presented in Table 1. Ten patients (43.5%) were emergently hospitalized for acute HF. The other patients were hospitalized to examine the etiologies and hemodynamic assessment of HF, and consider their treatment strategies. The etiologies of HF were as follows: dilated cardiomyopathy (*n* = 8, 34.8%); ischemic cardiomyopathy (*n* = 5, 21.7%); hypertrophic cardiomyopathy (*n* = 3, 13.0%); severe valvular heart diseases (*n* = 3, 13.0%); and other conditions, such as alcoholic cardiomyopathy (*n* = 1, 4.3%), cardiac amyloidosis (*n* = 1, 4.3%), tachycardia-induced cardiomyopathy (*n* = 1, 4.3%), and drug-induced cardiomyopathy (*n* = 1, 4.3%). Twelve patients (52.2%) had New York Heart Association (NYHA) functional class I or II at admission, while 20 patients (87.0%) had NYHA functional class I or II when the CT scan was performed (*p* = 0.052), which might be explained by intensive treatment for HF after admission. Similarly, the median levels of B-type natriuretic peptide (BNP) were significantly improved from initial admission to the day when the CT scan was performed (538 [259–1161] vs. 380 [151–537] pg/mL; *p* = 0.003). Atrial fibrillation was present in seven patients (30.4%), and 13 patients (56.5%) had chronic kidney disease (estimated glomerular filtration rate, < 60 mL/min/1.73 m^2^). On the day when CT was performed, none of the patients was given oxygen therapy or intravenous injection, and 18 patients (78.3%) were on oral diuretics.

**Table 1:** Patient’s clinical characteristics

Variable	*N* = 23
Age, y	60 (51–72)
Male, *n* (%)	16 (69.6)
Left ventricular ejection fraction, %	31 (26–53)
Diabetes mellitus, *n* (%)	8 (34.8)
History of smoking, *n* (%)	9 (39.1)
Profiles when CT was performed	
Body mass index, kg/m^2^	24.8 (21.3–27.6)
Systolic blood pressure (mmHg)	120 (90–131)
Heart rate, beats per minute	71 (58–81)
Oxygen saturation, %	97 (96–98)
NYHA functional class I or II, *n* (%)	20 (87.0)
eGFR < 60 mL/min/1.73 m^2^, *n* (%)	13 (56.5)
BNP, pg/mL	380 (151–537)
Oral medication when CT was performed	
ACEI/ARB, *n* (%)	17 (73.9)
Beta-blocker, *n* (%)	21 (91.3)
MRA, *n* (%)	13 (56.5)
Diuretics, *n* (%)	18 (78.3)
Right heart catheterization parameter	
Mean right atrial pressure, mmHg	3 (1–7)
Mean pulmonary artery pressure, mmHg	18 (12–26)
Pulmonary arterial wedge pressure, mmHg	9 (7–20)
Cardiac index, L/min/m^2^	2.7 (2.3–3.1)
Stroke volume index, mL/beat/m^2^	33.7 (25.7–46.4)

Data are described as median (25th–75th percentile)

Abbreviations: *ACEI*, angiotensin-converting enzyme inhibitor; *ARB*, angiotensin II receptor blocker; *BNP*, B-type natriuretic peptide; *CT*, computed tomography; *eGFR*, estimated glomerular filtration rate; *MRA*, mineralocorticoid receptor antagonists; *NYHA*, New York Heart Association

### Relationship between the SVC area and mRAP

**Fig. 2:**
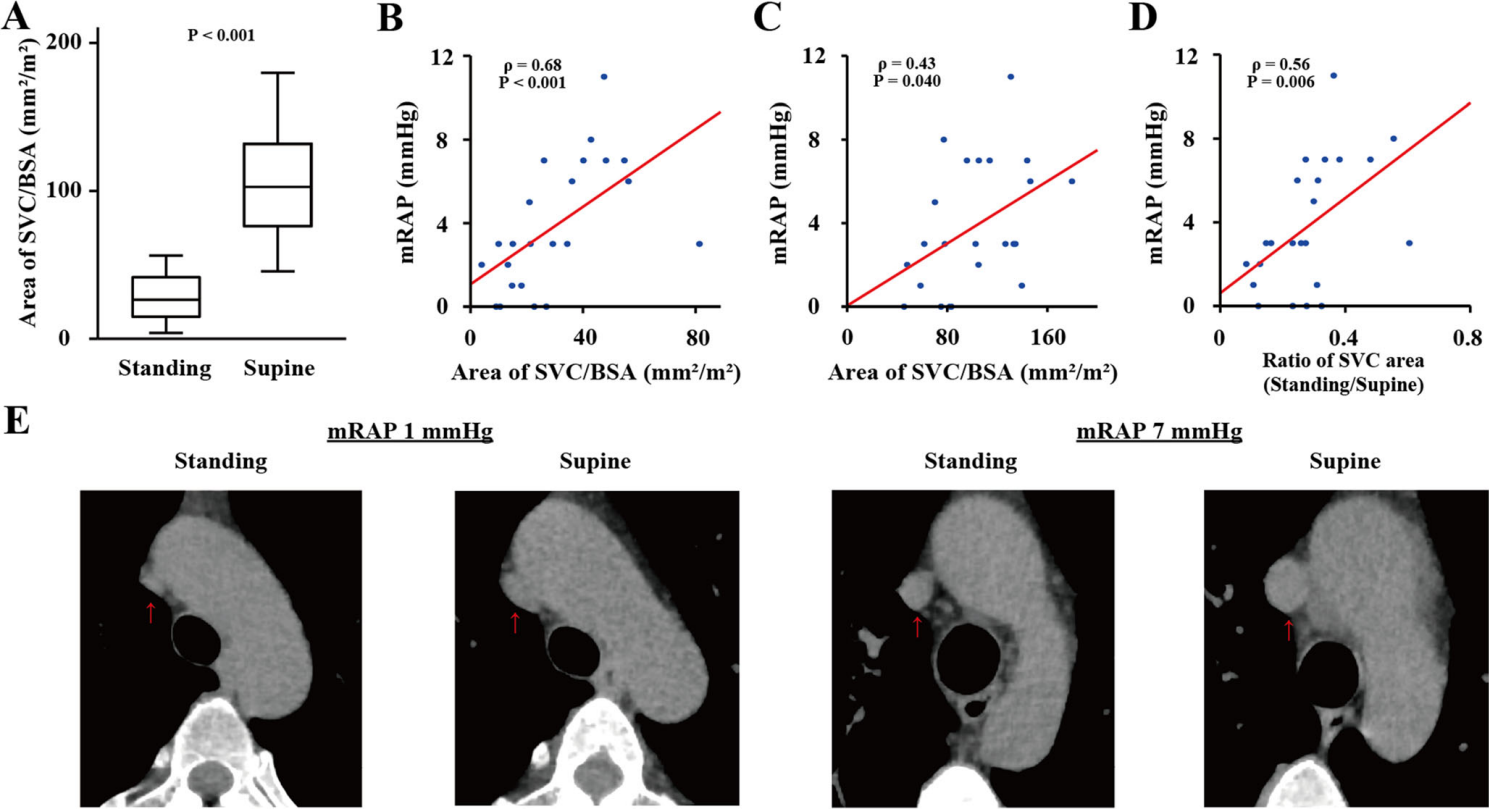
Computed tomography (CT)-based measurements of the superior vena cava (SVC) area. **A** Differences in the SVC area adjusted by body surface area (BSA) between standing and supine positions. **B** Scatter plot of the SVC area adjusted by BSA in the standing position vs. mean right atrial pressure (mRAP) obtained from right heart catheterization, including regression line. **C** Scatter plot of the SVC area adjusted by BSA in the supine position vs. mRAP, including regression line. **D** Scatter plot showing the ratio of the SVC area in the standing position to that in the supine position vs. mRAP, including regression line. **E** Differences in the SVC areas in CT scan images between standing and supine positions in two cases. In a patient with mRAP = 1 mmHg, the SVC area was 16 mm^2^ in the standing position and 69 mm^2^ in the supine position. Similarly, in another patient with mRAP = 7 mmHg, the SVC area was 98 mm^2^ in the standing position and 204 mm^2^ in the supine position. The red arrows show the SVC

The SVC area was significantly smaller when measured in the standing position than in the supine position (47 [23–74] vs. 172 [126–217] mm^2^, *p* < 0.001), regardless of adjustment for body surface area (BSA; 26 [15–43] vs. 103 [75–133] mm^2^/m^2^, *p* < 0.001, Fig. 2A). The median ratio of the SVC area in the standing position to that in the supine position was 0.27 (0.16–0.33).

The area of the SVC in both positions exhibited a moderate correlation with mRAP (*ρ* = 0.69, *p* < 0.001 in the standing position; *ρ* = 0.51, *p* = 0.013 in the supine position), regardless of adjustment for BSA (*ρ* = 0.68, *p* < 0.001 in the standing position, Fig. 2B; *ρ* = 0.43, *p* = 0.040 in the supine position, Fig. 2C). The ratio of the SVC area in the standing position to that in the supine position showed a moderate correlation with the mRAP (*ρ* = 0.56, *p* = 0.006) (Fig. 2D).

The area of the SVC adjusted by BSA in both positions exhibited a moderate correlation with BNP level (*ρ* = 0.62, *p* = 0.002 in the standing position; *ρ* = 0.47, *p* = 0.022 in the supine position). The correlation coefficients *ρ* between the area of the SVC adjusted by BSA in standing and supine positions and mean pulmonary arterial pressure (mPAP) were 0.37 (*p* = 0.083) and 0.59 (*p* = 0.003), respectively. The correlation coefficients *ρ* between the area of the SVC adjusted by BSA in standing and supine positions and pulmonary arterial wedge pressure (PAWP) were 0.40 (*p* = 0.060) and 0.42 (*p* = 0.044), respectively.

Figure 2E shows the differences in the SVC areas in CT scan images between standing and supine positions in two cases.

### Relationship between the IVC area and mRAP

**Fig. 3:**
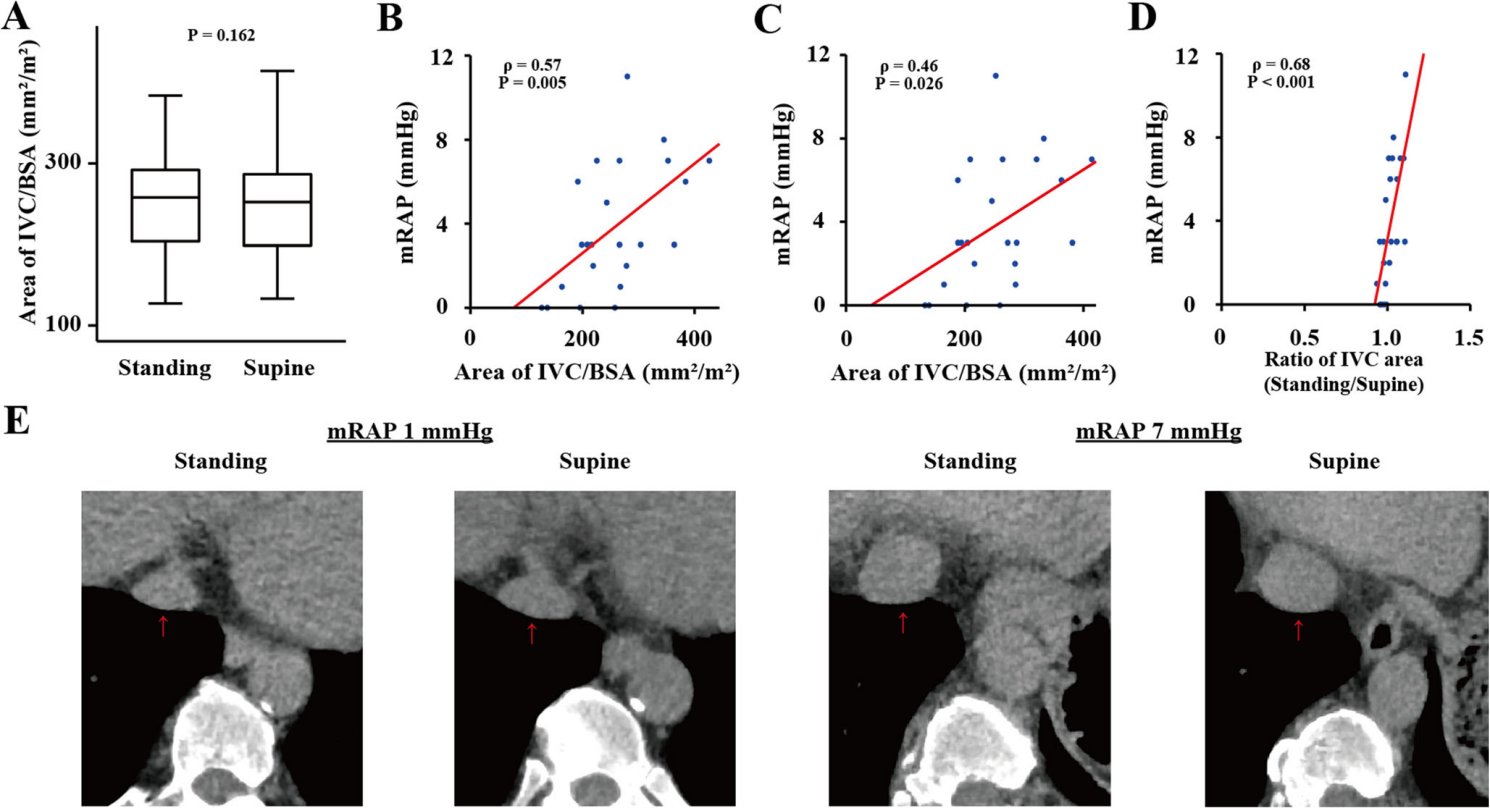
Computed tomography (CT)-based measurements of the inferior vena cava (IVC) area. **A** Differences in the IVC area adjusted for body surface area (BSA) between standing and supine positions. **B** Scatter plot of the IVC area adjusted by BSA in the standing position vs. mean right atrial pressure (mRAP) obtained from right heart catheterization, including regression line. **C** Scatter plot of the IVC area adjusted by BSA in the supine position vs. mRAP, including regression line. **D** Scatter plot showing the ratio of the IVC area in the standing position to that in the supine position vs. mRAP, including regression line. **E** Differences in the IVC areas in CT scan images between standing and supine positions in two cases. In a patient with mRAP = 1 mmHg, the IVC area was 193 mm^2^ in the standing position and 202 mm^2^ in the supine position. Similarly, in another patient with mRAP = 7 mmHg, the IVC area was 632 mm^2^ in the standing position and 575 mm^2^ in the supine position. Red arrows show the IVC

The IVC area was not significantly different when measured in the standing position as compared with that in the supine position (432 [356–541] vs. 420 [344–537] mm^2^, *p* = 0.148), regardless of adjustment for BSA (258 [199–304] vs 252 [194–287] mm^2^/m^2^, *p* = 0.162, Fig. 3A). The median ratio of the IVC area in the standing position to that in the supine position was 1.01 (0.98–1.06). In patients with mRAP ≤ 5 mmHg, the area of the IVC was also not significantly different between both positions (411 [351–464] vs. 409 [322–475] mm^2^, *p* = 0.532), regardless of adjustment for BSA (219 [196–268] vs. 216 [189–285] mm^2^/m^2^, *p* = 0.496), and the median ratio of the IVC area in two positions was 0.99 (0.96–1.02). However, in patients with mRAP > 5 mmHg, the area was significantly larger in the standing position (586 [398–648] vs. 540 [377–616] mm^2^, *p* = 0.012), regardless of adjustment for BSA (313 [236–376] vs. 292 [220–356] mm^2^/m^2^, *p* = 0.012), and the median ratio of the IVC area in two positions was 1.05 (1.02–1.09).

The area of the IVC in both positions exhibited a moderate correlation with mRAP (*ρ* = 0.57, *p* = 0.004 in the standing position; *ρ* = 0.52, *p* = 0.011 in the supine position), regardless of adjustment for BSA (*ρ* = 0.57, *p* = 0.005 in the standing position, Fig. 3B; *ρ* = 0.46, *p* = 0.026 in the supine position, Fig. 3C). The ratio of the IVC area in the standing position to that in the supine position showed a moderate correlation with the mRAP (*ρ* = 0.68, *p* < 0.001) (Fig. 3D).

The area of the IVC adjusted by BSA in both positions exhibited a strong correlation with BNP level (*ρ* = 0.76, *p* < 0.001 in the standing position; *ρ* = 0.73, *p* < 0.001 in the supine position). The correlation coefficients *ρ* between the area of the IVC adjusted by BSA in standing and supine positions and mPAP were 0.33 (*p* = 0.121) and 0.24 (*p* = 0.273), respectively. The correlation coefficients *ρ* between the area of the IVC adjusted by BSA in standing and supine positions and PAWP were 0.38 (*p* = 0.072) and 0.27 (*p* = 0.217), respectively. Figure 3E shows the differences in the IVC areas in CT scan images between standing and supine positions in two cases.

### Discrimination of the SVC and IVC areas for normal versus high mRAP

**Fig. 4:**
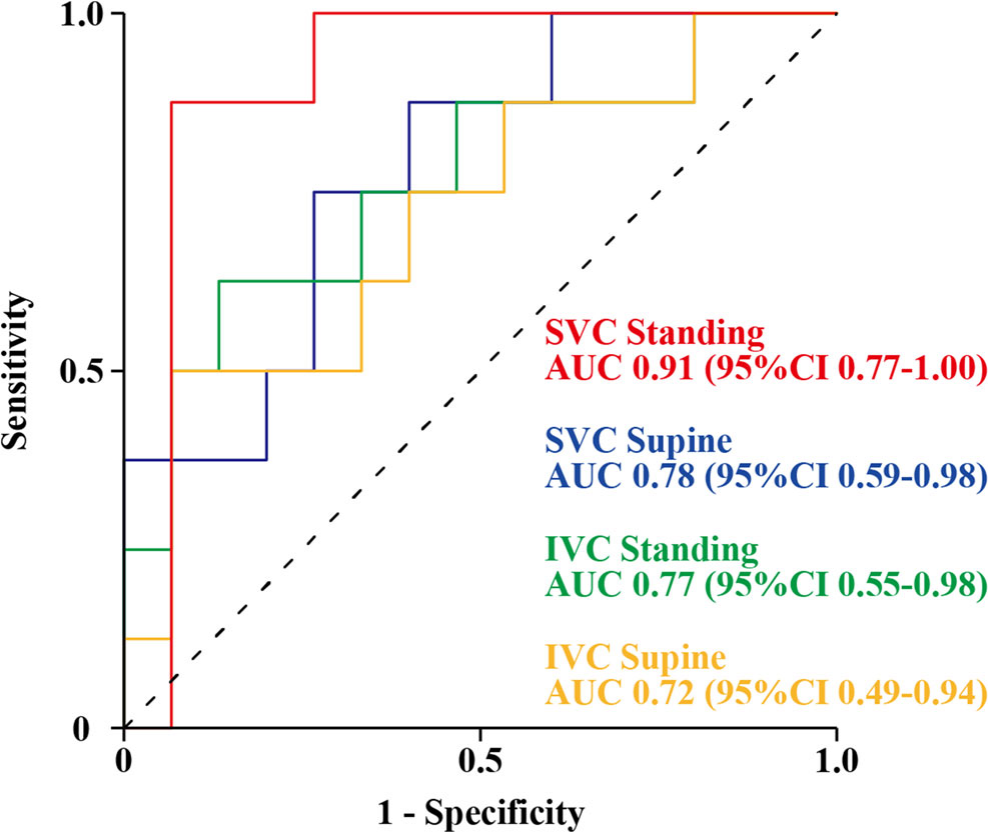
Receiver operating characteristic (ROC) curves. ROC curves with the superior vena cava (SVC) and inferior vena cava (IVC) areas adjusted by body surface area in both standing and supine positions for identifying mean right atrial pressure > 5 mmHg. Abbreviations: AUC, area under the receiver operating characteristic curve; CI, confidence interval

Using an mRAP cut-off point of 5 mmHg, the SVC area in the standing position showed the strongest discrimination, followed by the SVC area in the supine position and the IVC area in both positions (AUC, 0.90; 95% confidence interval [CI], 0.76–1.00 for the SVC area in the standing position; AUC, 0.81; 95% CI, 0.63–0.99 for the SVC area in the supine position; AUC, 0.78; 95% CI, 0.55–1.00 for the IVC area in the standing position; and AUC, 0.74; 95% CI, 0.50–0.99 for the IVC area in the supine position), regardless of adjustment for BSA (AUC, 0.91; 95% CI, 0.77–1.00 for the SVC area in the standing position; AUC, 0.78; 95% CI, 0.59–0.98 for the SVC area in the supine position; AUC, 0.77; 95% CI, 0.55–0.98 for the IVC area in the standing position; and AUC, 0.72; 95% CI, 0.49–0.94 for the IVC area in the supine position; Fig. 4). Nevertheless, no statistical difference was observed between each situation.

### Bland−Altman plots for upright versus supine measurements

**Fig. 5:**
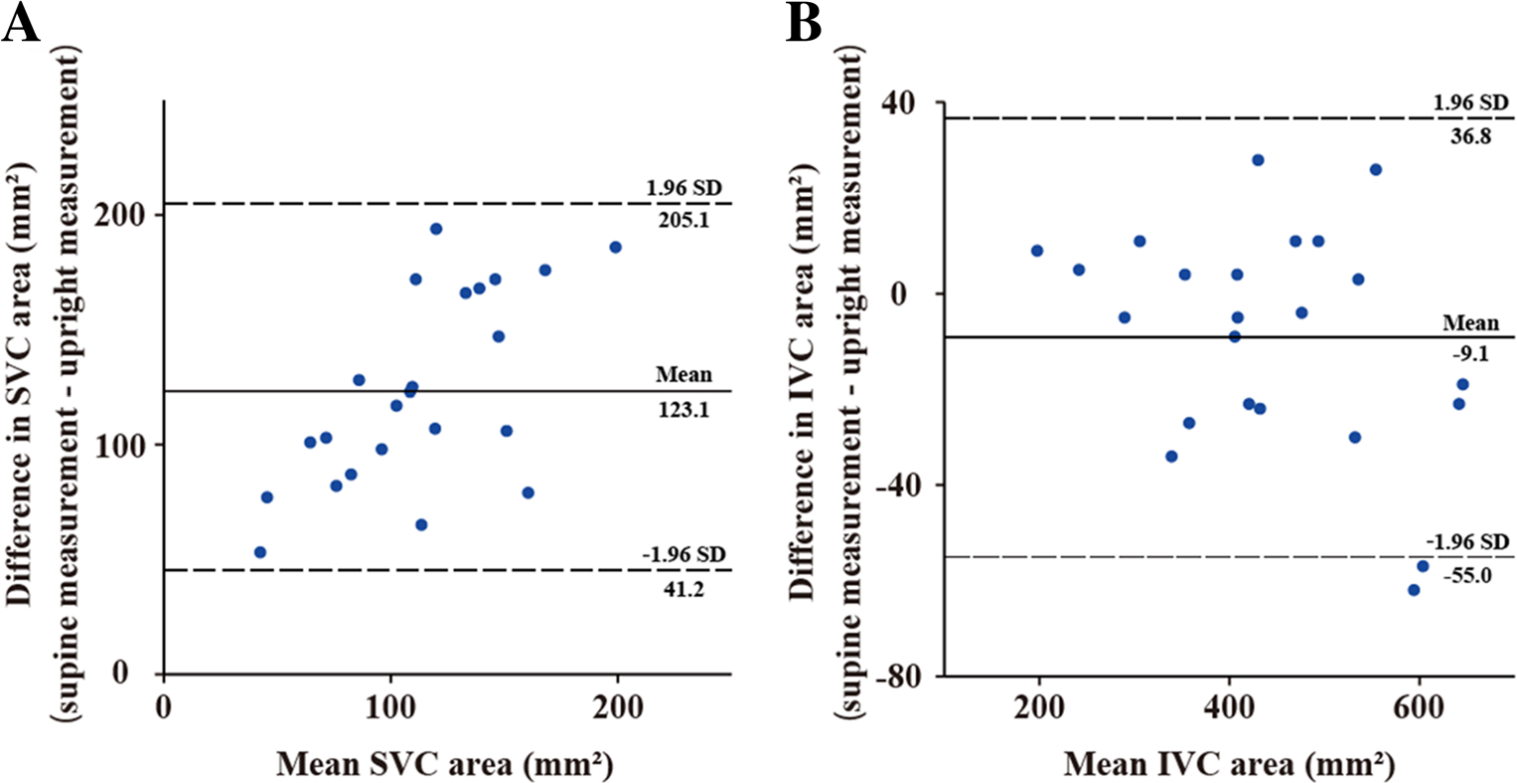
Bland−Altman plots. **A** Bland−Altman plot for superior vena cava (SVC) area demonstrates the calculated difference (supine measurement − upright measurement) on the *Y*-axis and the mean value from both measurements on the *X*-axis. Solid lines represent the mean value of the difference (supine measurement − upright measurement); dashed lines show the mean difference ± 1.96 standard deviation (SD). **B** Bland−Altman plot for inferior vena cava (IVC) area demonstrates the calculated difference (supine measurement − upright measurement) on the *Y*-axis and the mean value from both measurements on the *X*-axis. Solid lines represent the mean value of the difference (supine measurement − upright measurement); dashed lines show the mean difference ± 1.96 SD

Figure 5A, B show Bland−Altman plots for upright versus supine measurements of the SVC and IVC areas, respectively. The mean difference in the SVC area between supine and upright measurements was 123.1 ± 41.8 mm^2^ (95% CI, 105.0−141.2 mm^2^; limits of agreement, 41.2−205.1 mm^2^). The mean difference in the IVC area between supine and upright measurements was −9.1 ± 23.4 mm^2^ (95% CI, −19.3 to 1.0 mm^2^; limits of agreement, −55.0 to 36.8 mm^2^).

### Inter- and intraobserver agreements

The inter- and intraobserver agreements for all measurements on CT were substantial (0.962–0.991) (Supplemental Table 1).

## Discussion

In this study of patients with HF, we showed that the correlations between the standing position SVC and IVC areas and mRAP were stronger than those in the supine position, and the SVC area in the standing position showed the strongest accuracy in identifying patients with mRAP > 5 mmHg.

To the best of our knowledge, no study has evaluated the change in the area of the vena cava in patients with HF between upright CT in the standing position and conventional CT in the supine position. The previous study on healthy volunteers, as well as the present study on patients with HF, reported that gravity affects the vena cava area depending on positions; the SVC area was smaller in the standing position, but that of the IVC remained unchanged [1]. These results are consistent with a previous report that reported low hydrostatic pressure in the upper body and a gradual increase in the lower body [21]. Furthermore, the previous study demonstrated that the median ratio of the SVC area in the standing position to that in the supine position was 0.19 (0.15–0.28) in healthy volunteers [1], and the present study showed that this ratio was 0.27 (0.16–0.33) in patients with HF, which was also consistent with findings of a previous study that reported that patients with HF had high intravenous pressure in the upper body [22]. These findings suggest that upright CT, particularly upright CT-based measurements of the SVC area, has the potential to precisely estimate mRAP.

Most clinicians use non-invasive methods for estimating hemodynamic parameters by echocardiography in patients with HF [6, 7]. However, their estimation is limited to the heart and IVC, and the results are influenced by the sonographer’s skill and measurement error. Indeed, the IVC diameter and its collapsibility index which estimate mRAP can be misleading and limited, due to the often elliptical and irregular shape of the IVC and the variable angle in which the ultrasound depicts the vessel, making it difficult to capture its true dimensions [23]. In contrast, CT-based assessment enables accurate measurements of the comprehensive thoracic cardiopulmonary circulation system inside the body without the influence from body habitus or lung hyperinflation. Additionally, it can provide true orthogonal cross-sections of the comprehensive thoracic cardiopulmonary circulation system, including the SVC and IVC areas, which can lead to accurate estimation of mRAP. This study showed that upright CT may measure mRAP precisely with convenience, safety, good reproducibility, and good feasibility.

Physical diagnosis of jugular vein distension is an established clinical appraisal for venous congestion [24]; however, it is observer-dependent [25]. In addition, chest X-ray is used in routine clinical practice to diagnose HF; however, this diagnostic modality has low sensitivity, which can result in delayed detection of HF [26]. Some patients may have mildly worsened dyspnea with a slightly expanded cardio-thoracic ratio on chest X-ray scan but without apparent physical findings, such as leg edema or jugular vein distension. In such cases, upright CT can be a promising non-invasive tool for estimating mRAP. This may facilitate decision-making for optimal management strategies, such as temporary addition of diuretics, through direct detection of slight changes in the vena cava, especially in the SVC, which may prevent admission due to worsening of HF.

Some precautions should be taken into consideration when performing upright CT in patients with HF. In contrast to echocardiography, upright CT is difficult to perform in patients who are unstable (i.e., in those requiring non-invasive ventilation or intubation) because it requires the patients to maintain a standing position during scanning. However, we believe that the important clinical usefulness of upright CT is to identify exacerbation of HF in the early stages. Although patients need to maintain a standing/sitting position, this could be a more comfortable position for symptomatic patients with HF. Furthermore, we did not perform contrast CT. Although contrast-enhanced CT depicts vessels more clearly, we believe that it would be unlikely to add higher accuracy in measurements of the SVC and IVC areas. In addition, we avoided administration of contrast that would potentially affect the patients’ circulation status. However, further studies are needed to see if contrast-enhanced CT can safely and equally accurately measure SVC and IVC areas.

### Limitations

There were some limitations in the present study. As upright CT has been introduced only at our university-based hospital center at present, the sample size was small. The sample size was also influenced by our careful ethical considerations, regarding safety in particular. Second, patients were required to be able to maintain a standing position safely during CT, without oxygen therapy, which may have resulted in the enrolment of a study population with less severe HF conditions. Furthermore, some differences in practice exist between Japan and Western countries. In Japan, some patients are hospitalized to examine the etiologies and hemodynamic assessment of HF and consider their treatment strategies, which might have led to selection bias.

## Conclusions

Upright CT-based measurements of the SVC area are a feasible, easily applicable, and non-invasive method for evaluating mRAP precisely under the influence of gravity in patients with HF. Clinical management of patients with HF based on this modality may lead to early assessment of conditional changes and reduced hospitalization for HF exacerbation. Further prospective and multicenter studies to verify and disseminate the usefulness of upright CT are warranted.

## Supplementary Information

ESM 1 (DOCX 41 kb)

## Abbreviations

AUC: Area under the receiver operating characteristic curve
BSA: Body surface area
CI: Confidence interval
HF: Heart failure
IVC: Inferior vena cava
mRAP: Mean right atrial pressure
NYHA: New York Heart Association
RHC: Right heart catheterization
SVC: Superior vena cava

## Declarations

### Guarantor

The scientific guarantors of this publication are Yoshitake Yamada and Masahiro Jinzaki.

### Statistics and biometry

No complex statistical methods were necessary for this paper.

### Methodology

• prospective

• cross-sectional study

• performed at one institution
