# Supplementary material for: Estimating right atrial pressure using upright computed tomography in patients with heart failure
Source: Eur Radiol. 2022 Dec 28;33(6):4073–81. doi: 10.1007/s00330-022-09360-8 (PMC10182146; doi:10.1007/s00330-022-09360-8)
Supplement: Supplementary file 1 — (DOCX 41 kb) [file 330_2022_9360_MOESM1_ESM.docx]

**Supplemental Material**

**Supplemental Table 1.** Interobserver and intraobserver agreements for all measurements on computed tomography (CT) in the upright and supine positions.

|  | Interobserver agreement  (95% confidence interval) | | Intraobserver agreement  (95% confidence interval) | |
| --- | --- | --- | --- | --- |
|  | Upright | Supine | Upright | Supine |
| Area of the SVC | 0.984  (0.944‒0.996) | 0.962  (0.872‒0.989) | 0.989  (0.963‒0.997) | 0.981  (0.938‒0.994) |
| Area of the IVC | 0.980  (0.931‒0.994) | 0.976  (0.919‒0.994) | 0.990  (0.968‒0.997) | 0.991  (0.970‒0.97) |

Abbreviations: IVC, inferior vena cava; SVC, superior vena cava.
